# Cut and Paste: The Mexican Axolotl, Experimental Practices and the Long History of Regeneration Research in Amphibians, 1864-Present

**DOI:** 10.3389/fcell.2022.786533

**Published:** 2022-05-05

**Authors:** Christian Reiß

**Affiliations:** University of Regensburg, Regensburg, Germany

**Keywords:** history of biology, Mexican axolotl, regeneration, Julius Schaxel, Paul Wintrebert, experimental embryology, laboratory animals

## Abstract

The Mexican axolotl (*Ambystoma mexicanum*) is one of the most important models in contemporary regeneration research and regenerative medicine. This is the result of the long history of the species as an experimental and laboratory bred animal. One of many research questions investigated in the axolotl is regeneration. The species’ astonishing ability to regenerate tissues and entire body parts already became apparent shortly after the first 34 living axolotls had been brought from Mexico to Europe in 1864. In the context of their unclear status as larvae or adults and the mysterious transformation of some animals into an adult form, the Paris zoologist Auguste Duméril cut off the gills of several individuals in an attempt to artificially induce the metamorphosis. This produced the first reports on the animals’ regenerative powers and led to sporadic but continuous investigations. But it remained just one of the many phenomena studied in axolotls. Only at the beginning of the 20th century, regeneration became a more prominent aspect in the experimental investigations of axolotls. In experimental embryology, regeneration in axolotls was used in three different ways: it was studied as a phenomenon in its own right: more importantly, it served as a macroscopic model for normal development and, together with other techniques like grafting, became a technical object in the experimental systems of embryologists. In my paper, I will look into how the axolotl became an experimental animal in regeneration research, the role of practices and infrastructures in this process and the ways in which regeneration in the axolotl oscillated between epistemic thing and technical object.

## Introduction

In a text on the history of regeneration research, Frederick Churchill has coined the term “Gipfelsammler’s myopia” in reference to “[t]his dread disease [which] commonly afflicts historians of science, philosophy, art and other areas of high culture. Its new name parodies an alpinist’s term that refers to a single-minded attention to dramatic mountain peaks accompanied by total neglect of the surrounding hills and valleys that lend definition and meaning to the territory.” (Churchill 1991: 115).

Though a truism in today’s history of science, the challenge still is how to avoid this myopia. In my paper, I will present a long durée history of a prominent animal in regeneration research, the Mexican axolotl (*Ambystoma mexicanum*), as a way into those surrounding hills and valleys. I will use the scientific life of the axolotl to explore the field of regeneration research from 1864, when the axolotl was first brought to Europe, until today.^1^ By taking this panoramatic view, I want to make visible the dynamics between experimental infrastructure, experimental practices and the occurrence of life in the life sciences—life in the sense of living animals. Instead of exclusively focusing on the peaks, i.e., renowned researchers, historically important theoretical debates or influential research programs, I will use the history of regeneration research in the axolotl to show how regeneration research in one particular species unfolded over time.

I will present the unexpected and rather reluctant beginnings of studying regeneration in the axolotl. By revisiting the different moments in which regeneration was investigated since the first axolotls came to Europe in 1864, I will demonstrate how regeneration was used in different conceptual and experimental settings, how the Mexican axolotl was molded into a model for these processes and how this shapes regeneration research in this species until today. Beyond the example of the axolotl, this history offers a window into the history of the experimental practices of regeneration research and the respective experimental organisms more generally. I will highlight the ways in which regeneration in the axolotl became part of an experimental system or—in a broader perspective—how regeneration research in the axolotl became part of the larger experimental culture of experimental embryology in the life sciences in the 19th, 20th and 21st century (Rheinberger 1997; 2017). I will use the examples of the French embryologist Paul Wintrebert (1867–1966) and the German embryologist Julius Schaxel (1887–1943) to detail the ways in which axolotls became part of the experimental systems of laboratory biology that were forming since the 1880s. With France and Germany, they also represent the two countries with the most intense use of axolotls in research before 1945.

The axolotl is a particularly instructive example in this respect. The first living animals that came to Europe in 1864 were the founder generation of a population of hundreds of thousands of axolotls exclusively bred in laboratories, zoos and private aquariums. At least until 1914, all axolotls in Europe were direct descendants of the Paris animals. This makes them different from many other animals used in experimental zoology, which were mostly caught in the wild. We will see this in the case of many local amphibians used in comparative studies together with the axolotl. Like many other experimental or model organisms, the axolotl only gradually acquired its role (Ankeny and Leonelli 2019). But in contrast to them, axolotls were bred in captivity and particularly in laboratories since the arrival of the first living individuals in Europe in 1864 (Reiß et al. 2015). The long history of the axolotl and its gradual transformation from an object of natural history into an organism of the laboratory helps us to understand the ways in which experimental systems began to form in the life sciences and how living animals became part of them.

Focusing on the case of regeneration more specifically, a particular experimental system can be understood in relation to the larger experimental culture of experimental embryology. The axolotl turns from the object of epistemic interest in natural history to a part of the experimental system centered on regeneration as an epistemic thing. In the larger experimental culture, we see how regeneration—together with other surgical techniques like grafting—then becomes a technical object used to investigate other phenomena—the practices of cut and paste. For the history of regeneration research, following the axolotl helps us to understand the ways in which regeneration became experimentalized.

### The Unexpected Career of an Experimental Animal

In 1867, Auguste Duméril (1812–1870), a French herpetologist and professor at the “Muséum d’Histoire naturelle”, the natural history museum in Paris, published the first scientific papers on regeneration in the Mexican axolotl (Duméril 1867a; Duméril 1867b). He reported on a series of experiments he had made in 1866 at the “Ménagerie”, the museum’s zoological garden. Indeed, the paper was not so much on regeneration but on experiments Duméril had designed to study a different phenomenon. The axolotl’s mysterious transformation from an aquatic into a land-living form had fascinated him and the scientific world since 1865 (Duméril 1865a; Reiß et al., 2015).

In 1864, the first 34 living Mexican axolotls were brought from Mexico City to Paris as part of the global circulation of organisms in France’s imperial networks. The axolotls were initially not meant for science but for the zoo. But Duméril would use his six animals to investigate a question that he had inherited from his father: whether axolotls are adult animals or larvae. This question was discussed since Alexander von Humboldt (1869–1859) had sent the first preserved specimens to Georges Cuvier (1769–1832) in Paris at the beginning of the 19th century. Duméril’s first report on the axolotls, dated to 31 October 1864, was just one paragraph in a longer report on the “collection des reptiles” (Duméril 1865b). In this part of the “Ménagerie”, the zoo of the “Muséum”, reptiles, amphibians, fish and insects and other invertebrates were kept. Duméril was responsible for this marginal space that had difficulties competing for public and administrative attention and appreciation with the mammals and birds in the other parts. He offered a short description of the species with reference to its history in natural history and to Cuvier, whose judgment of the axolotls as larvae he confirmed. Six months later, things had already become more complicated. What once was a known fact had turned into “uncertainties regarding the true nature of these Batrachians” (Duméril 1865c: 765)^2^, as Duméril reported to the “Académie des sciences”. The axolotls had reproduced. This had overthrown Cuvier’s anatomical judgment that the axolotls were just larvae of another species of salamander. But larvae do not reproduce, only adult animals do.

The axolotls had changed due to the mobilization of the living animals in Mexico. Cuvier had worked with preserved females of unknown age and had drawn his conclusion based on his method of comparative anatomy. Duméril had received living animals, five males and one female. Keeping the axolotls alive and bringing them to reproduction was a considerable challenge in the middle of the 19th century. A year after their arrival, a report from the “Jardin d’acclimatation”, the other Paris zoo that had received the bulk of the axolotls from Mexico, stated with some pride that only one of the animals had died (Lavison 1865). In the same year, Duméril was able to report the successful reproduction to the “Académie”. One year later, he noted that the number of axolotls in the “collection” had increased to 800 individuals (Duméril 1866).

The difference in reproductive success can be explained by the knowledge, practical experience and the specialized facilities that Duméril had at his disposal at the “collection” (Reiß 2022). The “collection” was founded by Duméril’s father Constant Duméril (1774–1860) in 1838. He bought the animals and the equipment of the traveling animal show of Honoré Vallée (1807–unknown), whom he also hired as his assistant. The “collection” was located in the abandoned monkey house of the “Ménagerie” and together with Vallée, Constant Duméril and later his son Auguste developed the space into a sophisticated facility to keep reptiles, amphibians, fish and insects from all over the world. They were kept in cages, aquariums and basins that were heated with an advanced indirect heating system. They put a lot of effort in observing the animals and their reaction to the new environment, figuring out the proper way to feed them and adapting their treatment accordingly.

With the living axolotls, Duméril seemed to have finally solved the question. He observed their reproduction in their aquatic, larvae-like state and concluded that they must be the adult form. But the axolotls had another surprise in store. Several individuals of the offspring of the first six animals started to transform. They turned into lung-breathing terrestrial amphibians; they went from water onto land. Instead of comparative anatomy, Duméril used animal husbandry as his method. His success also depended on the ability of the axolotls to adapt to the environment provided by him. He would continue to use this approach in his attempt to answer the questions that resulted from the axolotls’ transformation. What was this transformation of an apparently adult animal into something different? And why did only some animals transform? Was it a kind of metamorphosis, was it an irregular development or was it even an evolutionary event? Speculations and hypotheses flourished 6 years after the publication of Darwin’s “On the Origin of Species” (Darwin 1859).

In Paris, the axolotls had undergone multiple transformations. They changed from larvae to adult animals, they transformed from an aquatic form into a terrestrial form and they turned from an object of epistemic interest in natural history into a productive colony of living animals. Their productivity exceeded both the collection of preserved animals in the natural history museum and the stock of living animals in the closely connected zoo. But they very much fitted the demands of embryological research, where large numbers of slightly differing embryonic stages were used to trace the development of anatomical structures. They also provided a number of phenomena like the selective metamorphosis that called for further investigation that would make use of the availability of living animals. In the context of this research, regeneration was at first only a side effect. But the proliferation of the axolotls together with the advent of experimental zoology would soon bring the phenomenon to greater prominence. This approach necessarily covers a variety of definitions of regeneration or even the lack of explicit ones. Rather, the availability of living animals in large quantities and the questions at hand led to a gradual formation of experimental research.

### From Tentative Beginnings to an Experimental Culture

Duméril immediately thought about an empirical investigation. To better understand the cause of the transformation, he came up with two experimental approaches (Reiß 2022). Following his observations, he concluded:

“The atrophy of the branchial tufts and their gradual disappearance being the first signs of the metamorphosis which is going to take place, I have endeavored to provoke a change in the mode of respiration by obliging the animals to make use of their pulmonary organs. I made at first some fruitless experiments, consisting partly in gradually diminishing the quantity of water in which the axolotls were kept, so as to leave them, after a certain time, nothing but a layer of damp sand, and partly in arranging in their aquarium a broad shelter, which enabled them to live alternately immersed and out of the liquid. To obtain any result there was another experiment to be made. It was necessary to destroy the branchiae, in order to ascertain whether, when rendered compulsorily animals with a pulmonary respiration [sic], the axolotls would undergo the modifications which I have enumerated.” (Duméril 1867: 447).

The first experimental approach was to manipulate the living conditions of the animals to force them onto land. He would slowly lower the water level in an aquarium or offer a shore-like scenario. The second approach was complementary and aimed at the animals’ respiratory organs. Duméril performed a series of experiments where he would cut the external gills of the axolotls. Similar to the first approach, this had no effect on the animals. But Duméril could conclude that axolotls have enormous regenerative abilities, similar to other urodelous amphibians, but of a greater magnitude.

These first experiments on regeneration in axolotls were not done for studying regeneration. Rather, it was a well-known trait of amphibians and reptiles. Already Constant Duméril had dedicated a few pages on “la reproduction des membres” (Duméril and Bibron 1834: 206)—the reproduction of extremities—in the first volume of his comprehensive “Erpétologie générale, ou, Histoire naturelle complète des reptiles”.^3^ In this passage that is part of the chapter on the nutrition of reptiles, he cites the Roman polymath Pliny (AD 23/24–79) and the German anatomist Johann Friedrich Blumenbach (1787–1840) as the authorities on the topic, describes the phenomenon together with the literature, and finally reports his own experiences. These also include experiments in which he cut off body parts of several species of mostly amphibians. The results are stated but no further explanation is attempted. In the tradition of natural history, Duméril listed regeneration as a trait of the taxon that did not call for an explanation in the physiological sense.^4^

In the same way, his son Auguste understood regeneration in the axolotl as an effect that was either helpful in healing the animals after surgery or detrimental in cases in which he wanted to deprive the animals of their gills for a longer period—he had to keep cutting them. The opportunity to perform such experiments came with the availability of the axolotls and his success in breeding them.

Both experiments did not induce a transformation, but they set the direction for further research in the axolotl. Many of Duméril’s numerous articles on the axolotls in Paris were translated into other languages and the knowledge of the axolotls and of their regenerative abilities began to circulate. But also the animals themselves spread across Europe (Reiß 2020). Duméril was very successful in breeding them in the “collection”. Soon many naturalists and zoologists, but also zoos and aquarium enthusiasts had their own axolotls. They came either from Duméril directly or from the growing number of axolotl breeding colonies. The rising popularity of the aquarium was central in this process. Axolotls were the first non-native species for it. The spread of animals especially in anatomy and zoology institutes in the German-speaking world made them into one of the first non-domestic animals bred entirely in the laboratory (Reiß et al. 2015).

**FIGURE 1:**
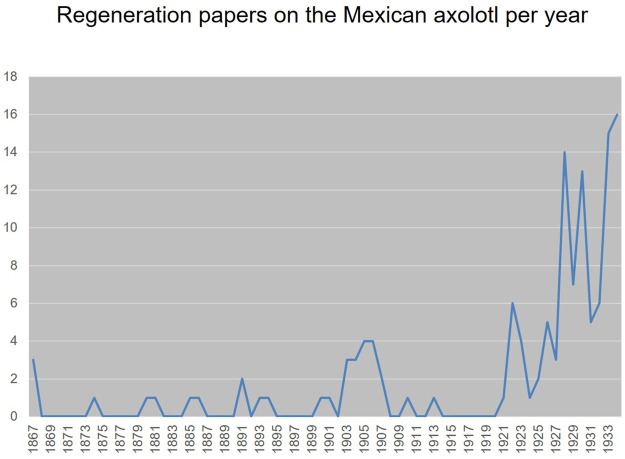
Papers on regeneration in the axolotl from 1864 to 1933. For the data basis, see Reiß (2020).

In their research, zoologists and anatomists used axolotls as a generic resource in descriptive embryology (“Entwicklungsgeschichte”) and comparative anatomy to supplement their material. In comparative studies, axolotls were either added as another amphibian species or they were used as the sole representative of the entire group. However, the use of experiments and thus the need for living animals was on the rise. The easy availability of the lab-bred axolotls made them into an obvious experimental object. Regeneration was one of many questions investigated in this context. But it took until the 20th century to have regeneration research in the axolotl really take off (Figure 1). Between 1867 and 1914, only 35 papers on regeneration in the axolotl were published—which makes it 0.7 papers per year. In contrast to that, from 1914 to 1933, 82 publications can be found, i.e., 4.3 papers per year.

**FIGURE 2:**
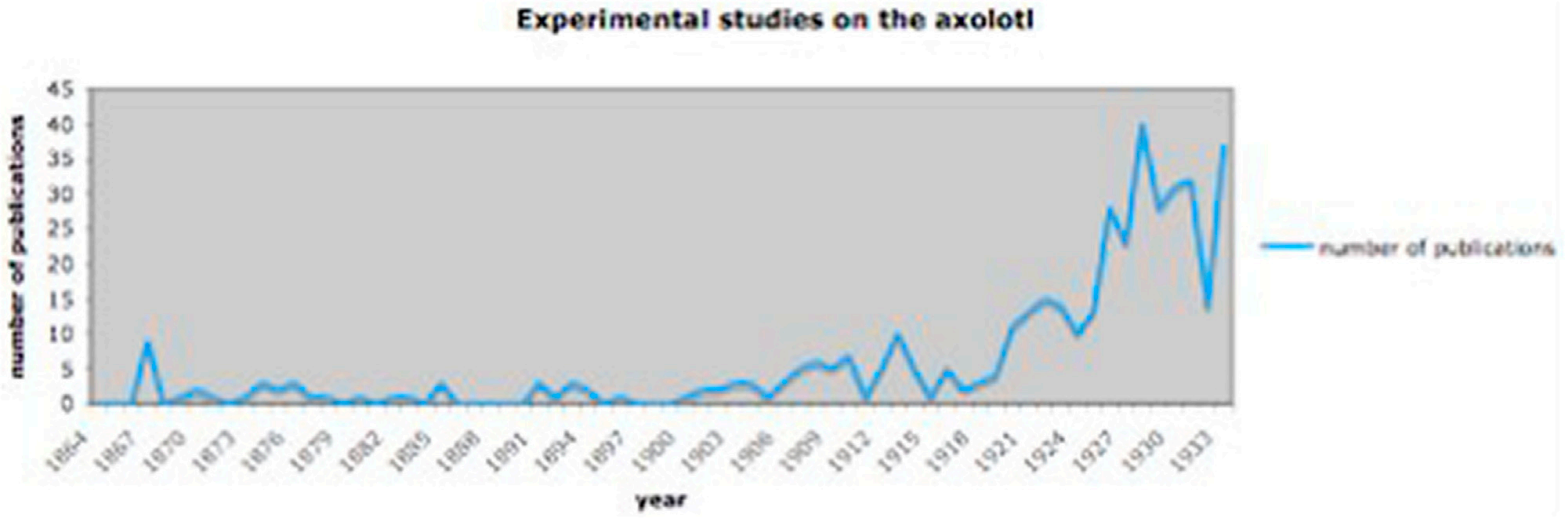
Papers on experiments using the axolotl from 1864 to 1933. For data, see Reiß (2020).

**FIGURE 3:**
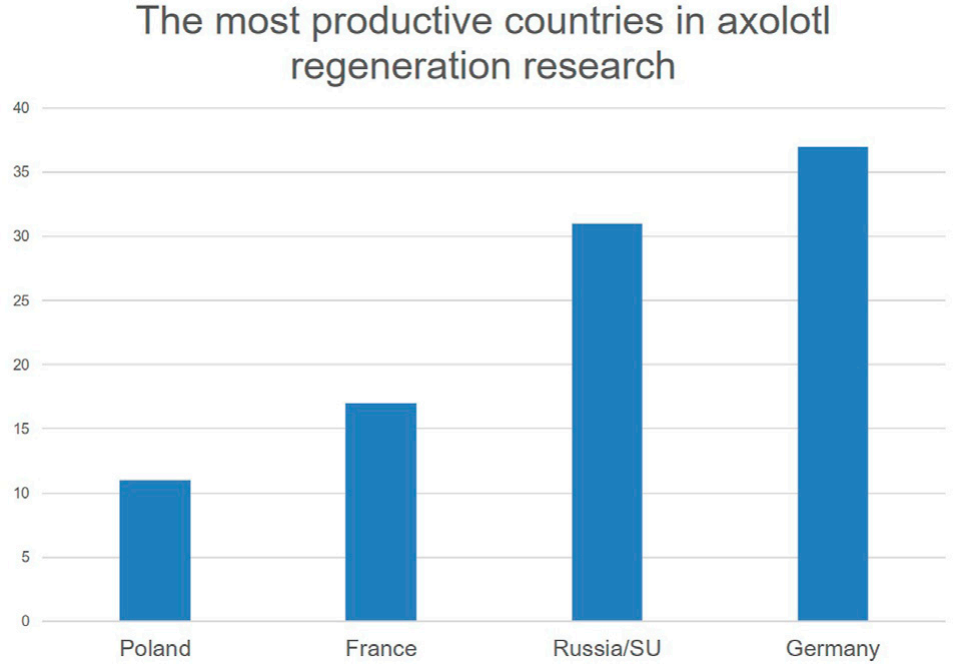
Regeneration research on the axolotl by country from 1864 to 1933. Please note that I assigned the papers to the territorial status at the time of their publication. For data, see Reiß (2020).

Compare this with the general development of publications on the axolotl, where the most substantial increase in experimental studies can be found from 1900 onwards (Figure 2). It shows that the most productive period of regeneration research in the axolotl was in the interwar period. The centers of regeneration research in the axolotl were Poland with 11, France with 17 papers, Germany with 37 papers and Russia/Soviet Union with 31 papers (Figure 3).

By the 1910s, we see a shift from the comparative embryology of the 19th century centered around morphology and taxonomic relations, to experimental zoology and mechanisms. In this shift, the regenerative ability in the axolotl and other species became more and more important while their taxonomic status was marginalized. The structure of three textbooks on regeneration illustrates this shift. Jean Nicolas Demarquay (1874–1875), who had worked together with Auguste Duméril on other topics, published his “De la régénération des organes et des tissus” in 1874 (Demarquay 1874). He divides his treatment of regeneration up into a part on animals of “lower organization” and a part on animals of “higher organization”. The latter part is then structured according to organ, body part and tissues. In 1909 the Austrian zoologist Hans Przibram (1874–1944) published a literature review on regeneration as the second part of this seven volume series “Experimental-Zoologie” (Przibram 1909). Although one of the founders of the “Biologische Versuchsanstalt” in Vienna and an early proponent of a general biology, his book is still organized taxonomically. The third example is Dietrich Barfurth’s (1849–1927) contribution to Emil Abderhalden’s massive “Handbuch der biologischen Arbeitsmethoden” from 1923 (Barfurth 1923). Barfurth begins his text with an introduction to experimental methods. The structure of the main part of the text strictly follows regeneration as a phenomenon. Instead of taxonomic groups, he differentiates between regeneration in embryos and regeneration in adult animals. The latter category is then divided up into a part on types of regeneration, one on its specific characteristics and one on the role of internal and external influences. Here, the different animal species are only interesting as carriers of particularly prominent forms of certain aspects of the phenomenon regeneration as a whole.

The three books also show how the understanding of regeneration changed and how a different epistemology began to form. Auguste Duméril and also Demarquay stood in the tradition of natural history. Here regeneration was understood as a trait of particular species and its different instances were compiled by the natural historian as part of a natural history of a particular taxonomic group as in Constant Duméril’s “Erpétologie générale”. Demarquay already set the focus on the phenomenon and tracked its appearance through the animal kingdom. Regeneration was closely tied to the concept of generation. Generation combined what we today understand as reproduction, development, heredity and evolution (Müller-Wille and Rheinberger 2014). In Przibram and Barfurth, generation was in the process of differentiation and regeneration became closely attached to development.

This gives an idea of how research in a particular phenomenon in a particular species took shape from the middle of the 19th century until the 1930s. The general increase in publications and the growing importance of experimental and laboratory studies reflect the general developments in the life sciences. The axolotl had turned into an easily available research organism for all sorts of experimental studies, regeneration being just one.

The cases of the French and German experimental embryologists Paul Wintrebert and Julius Schaxel, respectively, further illustrate this development. The cases are from two of the countries with the strongest research output on the axolotl and on regeneration in the axolotl (Figure 3). They help to understand the ways in which axolotls became experimental animals in regeneration research and the wider methodological and theoretical contexts of regeneration research.

### Regeneration Research in French Neo-Epigenetics and Neo-Lamarckism

The French embryologist Paul Wintrebert (1867–1966) began his research on axolotls at the beginning of the 20th century. After his degrees in medicine and natural sciences, he started to work at the laboratory of Frédéric Houssay (1860–1920) at the “École normale supérieure” (ENS) in Paris (Bounoure 1925; Fischer 1990). Houssay’s example shows the multiple research contexts and practices in which the axolotls were already embedded before their use as experimental animals. Before coming to the ENS, Houssay had been in Lyon, where he had already been working with axolotls. He used the animals to study embryogenesis together with Eugène Bataillon (1864–1953) (Houssay and Bataillon 1888a; Houssay and Bataillon 1888b). They used zygotes and early embryonic stages of axolotls to study cleavage and gastrulation, specifically the formation of the mesoderm and the *chorda dorsalis*.

Houssay discusses the advantages and problems of axolotls for embryological investigation in some detail (Houssay 1893: 3). For him, the major disadvantage was that—similar to other amphibians—already the yolk of the axolotl egg is pigmented. The resulting opacity of egg and embryo made observation and manipulation difficult.^5^ For Houssay, this was outweighed by the comparably large embryos and the high reproduction rate of the animals. With only two or three breeding couples, he was able to get enough embryos to have twelve embryos available twice every day. Though these embryos looked very similar, they showed small differences in their development upon closer inspection. This made it possible to construct an almost continuous series of developmental stages, from which microscopic preparations for the structures of interest could be made. Through the mass of embryos a much greater resolution of the processes of early development became possible.

Even though Houssay did not refer to it explicitly, this relates to a method initiated by German zoologist Carl Gottfried Semper (1832–1893) in 1878 to manipulate the reproduction of axolotls and to have fertilized eggs all year long (Semper 1878). Similar to Semper, who worked closely with aquarium fanciers in Germany (Reiß 2012: 323), Houssay relied on the knowledge and the practices of aquarium hobbyists—“les éleveurs d’Axolotl” (Houssay 1890a: 147)—to develop this method. Houssay’s use of the axolotl shows how the animal was turned into a laboratory tool even before experimental investigations began.

After his move to Paris in 1886, Houssay continued his embryological studies with axolotls (Houssay 1889; Houssay 1890a; Houssay 1890b; Houssay 1891; Houssay 1892; Houssay 1893; Houssay 1894). He now focused on the development of the vertebrate skull. The study of this problem in the axolotl had a history that predated the arrival of the living animals in 1864. The German anatomist Carl Gegenbaur had begun to study the development of the vertebrate skull using axolotl specimens already as a student of Albert Kölliker in Würzburg in 1849 (Friedereich and Gegenbaur 1849; Olsson and Hoßfeld 2003). In the course of his career as one of the most eminent morphologists of the 19th century, he developed his theory of head metamerism (Gegenbaur 1888; Mitgutsch 2003; Depew and Olsson 2008). His theory that the vertebrate skull consisted of a segmented and a non-segmented part was contested by the German zoologist and founder of the Naples Zoological Station, Anton Dohrn, who argued for a fully segmented skull (Di Gregorio 1995). Houssay followed Dohrn’s position that the entire vertebrate skull derived ontogenetically and phylogenetically from branchial arches (Houssay 1900).

Houssay’s laboratory at the ENS was the place in which Paul Wintrebert got to know axolotls as laboratory animals and where he learned to work with them. Between 1903 and 1928, he worked intensely with the animals. In his research he was particularly interested in regeneration and metamorphosis in amphibians. He worked with animals from Houssay, but also received some of his axolotls from Léon Vaillant (1834–1914), Duméril’s successor at the “Muséum” (Wintrebert 1907: 521).

Wintrebert studied the two phenomena experimentally in the axolotl and other amphibians. His regeneration experiments were a reaction to the results of the Breslau embryologists Alfred Schaper (1863–1905) (Schaper 1898) and Kurt Goldstein (1878–1965) (Goldstein 1904), as well as to the results of Richard Rubin (Rubin 1903), who did his dissertation with Barfurth in Rostock. All three claimed that their experiments on various amphibians showed that the influence of the nervous system on ontogenesis and regeneration was increasing with the age of the organism. Only during embryogenesis, both processes were independent of the nervous system and governed by an “immanente Energie” (Goldstein 1904: 105)—an energy or drive from within the organism.

It was Barfurth, who had promoted the amphibian egg and particularly the one of the axolotl as a new object for experimental embryology (Barfurth 1893). In the last decades of the 19th century, the marine stations along the European coasts were the centers of zoological research, particularly the Naples Zoological Station (Fantini 2000; De Bont 2015). The investigation of marine invertebrates did not only have a major influence on morphological, embryological and phylogenetic questions. The almost direct access to the living animals also brought about a shift to more and more experimental forms of research. For zoologists like Barfurth, who was at that time at Dorpat university, the financial and practical efforts necessary made regular visits and thus also experimental research impossible (Reiß 2012). Furthermore, the fact that the visits were only possible during the semester breaks posed additional difficulties. Switching from marine organisms to amphibians offered a solution to many of these problems. With the axolotl, there was even an organism already at home in the laboratory and readily available. Based on Semper’s method for regularly producing axolotl eggs, Barfurth developed a research program on regeneration in vertebrates, in which he mostly used amphibians as research organisms (Barfurth 1906; 1923).

In the research that Wintrebert reacted to, Rubin had used *Rana fusca* (today *Pelobates fuscus*) and axolotls, while Schaper and Goldstein worked with *Rana esculenta* (the Edible frog, today *Pelophylax esculentus*). Wintrebert did his experiments with *Alytes obstetricans* (the midwife toad), *Rana temporaria* (the common frog) and axolotls. The choice of organisms reflects the comparative tradition of embryology from which this research emerged, but also the pragmatic choice governed by availability and practicability.

For his experiments, Wintrebert made use of the axolotls that he had learned to produce in Houssay’s laboratory. The other amphibian species were most likely collected in the field. While the selection of animals still shows the comparative tradition of embryology, the experimental approach developed its own dynamics, particularly with the axolotls. Wintrebert began with multiple animals paying close attention to the anatomical details during surgery and carefully observing the regenerative processes. He would also vary the procedure slightly in different animals and thus create controls for his experiments (e.g., Wintrebert 1904b: 725).

From his results, he concluded that ontogenesis and regeneration are independent of the nervous system at any point in the life of the organism (Wintrebert 1903a; 1903b; 1904a; 1904b; 1904c; 1905a; 1905b; 1905c; 1906b; 1906c; 1906d). From there, he pursued investigations of metamorphosis as another developmental phenomenon. He first transferred his previous approach and investigated a potential influence of the nervous system on metamorphic processes (Wintrebert 1905d). He then extended his research on the phenomenon and investigated the influence of various environmental factors (Wintrebert 1906a; 1907; 1910a; 1910b) and tried to control it experimentally (Wintrebert 1908a; 1908b; 1908c; 1909).

This research took up the approaches first attempted by Duméril and later successfully applied by the German naturalist Marie von Chauvin (1848–1921) (Geißler and Reiß 2021). Similar to Chauvin and the much more explicit Paul Kammerer (1880–1926), who also followed up on her experiments, Wintrebert was an outspoken neo-epigeneticist and neo-Lamarckist (Fischer 1990; Persell 1999). In contrast to Schaper, Goldstein and Rubin, who referred to an inborn energy as an explanation, he highlighted the importance of the processuality of development and the crucial role of environmental factors in it. He saw ontogenesis and evolution connected as active responses of the orgasim to the environment. He would develop a biochemical Lamarckism to solve the problem of finalism that he saw as the problem at the heart of neo-Darwinism (Wintrebert 1962; 1963; Boesiger 1974: 30). His findings in the axolotl were taken up by Pierre Kropotkin in his writings on animals and their environment (Kropotkine 2015).

### Regeneration Research in German Experimental Embryology and Theoretical Biology

Similar to Wintrebert, the German embryologist Julius Schaxel was critical of both mechanistic preformation theories and of neo-Darwinian evolutionary theory and saw regeneration as a way to empirically criticize the orthodox framework of biological theory. For him, ontogeny was the basis to understand the organism as the central unit of life and a new basis for biology as a science. Schaxel would not adopt a Lamarckian position. He would only criticize what he called the historical conception of life, i.e., that evolution and the historicity of organisms are the theoretical basis for all of biology (Schaxel 1919).

Schaxel’s case gives a different perspective on the way of the axolotl into regeneration research. While in Wintrebert’s case, there is still a direct connection to Duméril’s axolotls and Paris, Schaxel started his research on regeneration in axolotls in 1918. At this point, both experimental embryology and the use of axolotls as laboratory animals can already be considered as established. Therefore, Schaxel chose the axolotl rather consciously as an experimental animal.

Schaxel was the last student of the German evolutionary biologist Ernst Haeckel. Like other Haeckel students, e.g., Wilhelm Roux and Hans Driesch, Schaxel became critical of Haeckel’s phylogenetic approach and turned to other questions, methods and objects of study. This was not just due to the speculative nature and the popular appeal of Haeckel's phylogeny but also part of the broader development towards a new foundation for biology as a scientific discipline. For his embryological research, Schaxel would regularly travel to the Naples Zoological Station and to other marine stations at the European coasts to collect and work on material (Reiß et al. 2007). With the outbreak of the First World War, many of the marine stations became inaccessible for German zoologists and Germany's defeat did not promise a quick return to the situation that had existed before 1914. Schaxel had been appointed associate professor at Jena university in 1916. He spent the war time with mostly theoretical studies and was looking for a way to work experimentally again in Jena. The zoological institute was not an option. Ludwig Plate, Haeckel’s successor, was as much of an enemy of Schaxel as he was of experimental zoology. With the help of a circle of Haeckel’s friends, Schaxel managed to win a grant from the Carl Zeiss Stiftung, the foundation of the local manufacturer of optical instruments. On 1 January 1918, he was able to formally open the “Anstalt für experimentelle Biologie”, his own research institute. The terms “experimental” and “biology” indicate his demarcation from what was happening in the zoological institute and other places at Jena university. In contrast to the descriptive investigations of morphological and phylogenetic structures in animals, he saw himself as part of a new generation of researchers who used experimental approaches to study the laws and mechanisms of life.

For the research after his dissertation, Schaxel began to shift from a descriptive embryology that was also supported by Haeckel to a fully experimental approach. For his second book, he studied various developmental processes in marine invertebrates. The first two parts of this multi-volume publication followed the descriptive agenda (Schaxel 1912; 1913). The third part shows the shift to experimental research when Schaxel began to manipulate the eggs of starfishes and annelids (Schaxel 1914). Here, he also made his first experiments with regeneration.

Since the organisms he had been working with in the time before the war were not accessible anymore, Schaxel had to look for a new research organism. He decided to work with the Mexican axolotl (Schaxel 1921a: 15). Schaxel explained his choice with a number of advantages the axolotl had. First and foremost, it was the regenerative capabilities of the species that attracted his attention. But he also listed more practical considerations. Their pervasiveness made axolotls readily available. Their long history as laboratory animals—at that time, axolotls had already been in European aquariums and laboratories for more than 50 years—promised no additional work in introducing the animals into the laboratory and the easy establishment of a large husbandry. He could already rely on established methods for keeping them and working with them experimentally, like the ones by Semper, Houssay and Barfurth. He specifically pointed to the fact that he wanted to do histological and cytological investigation and axolotl tissue was already well known for being easy to grow in culture. Schaxel thus could rely on the co-adaptation of the animals and the laboratory environment, also known as “generative entrenchment” (Griesemer 1992: 52).

How well established axolotls already were is shown by the fact that Schaxel could open his new institute on 1 January 1918. Even though the First World War was still raging, he was immediately able to start with his research. He was interested in regeneration in general and as a phenomenon that was central to embryological research. For Schaxel, it was the attempt to reinvent himself as an experimental biologist, but also a point of entry into the theoretical foundations of embryology that he wanted to reform with his research. During the First World War, Schaxel had intensified his philosophical interests. He turned the conceptual discussions that had been part of his publications into a central aspect of his research program for some time. From 1914 to 1917, he led a controversy with the former embryologist and vitalistic philosopher Hans Driesch on the justification of the latter’s “Entelechie” and the “harmonisch-äquipotentielles System” as useful concepts for biological research (Reiß 2007). The extended fourth part of his second book was published independently as a monograph in 1915 (Schaxel 1915). It offered a synthesis of the results of the first three parts and included an extended reflection on the conceptual problems of biology. In 1919, he published an analysis of the conceptual foundations of the different strands that made up biology and the problems that arose from their various inconsistencies (Schaxel 1919). In his “Anstalt” experimental research and theoretical reflection should go hand-in-hand to develop a robust conceptual framework for biology that would guide further research (Reiß 2007).

For Schaxel, regeneration was a central phenomenon in all this. It played “a major or even the central role in all theoretical considerations of contemporary biology” (Schaxel 1921a: 1). The question whether regeneration was re-generation, “Wiedererzeugung” in Schaxel’s words, or an entirely new generation, “Neubildung”, for him was linked to the question whether the regenerated organ was “typical” or “atypical” (Schaxel 1921a: 77). The theoretical centrality of this question was based in the epistemic specificity of biology as a science. Even though the life sciences had successfully adopted a mechanistic research program in the 19th century, the phenomenon of life did not stop to question the appropriateness of the causal method. Namely, the time around 1900 saw a renaissance of non-mechanistic explanations, most famously the organic philosophy of Hans Driesch, and attempts to solve the dichotomy between mechanism and vitalism via a third way. Schaxel was one of the most active organizers of these discussions in the German speaking world and a proponent of organicism as a solution to the foundational crisis of biology. Regeneration was central in this respect as it allowed the repeated experimental investigation of the phenomenon of regulation that was seen as the basic function of the organism. It thus allowed for a theoretical and empirical investigation of the limitations of the causal method in biology—Entwicklungsmechanik or causal morphology in this case. As it was a general phenomenon present in all organisms, it was no problem for Schaxel to switch from marine invertebrates to a neotenic salamander as a research organism.

**FIGURE 4:**
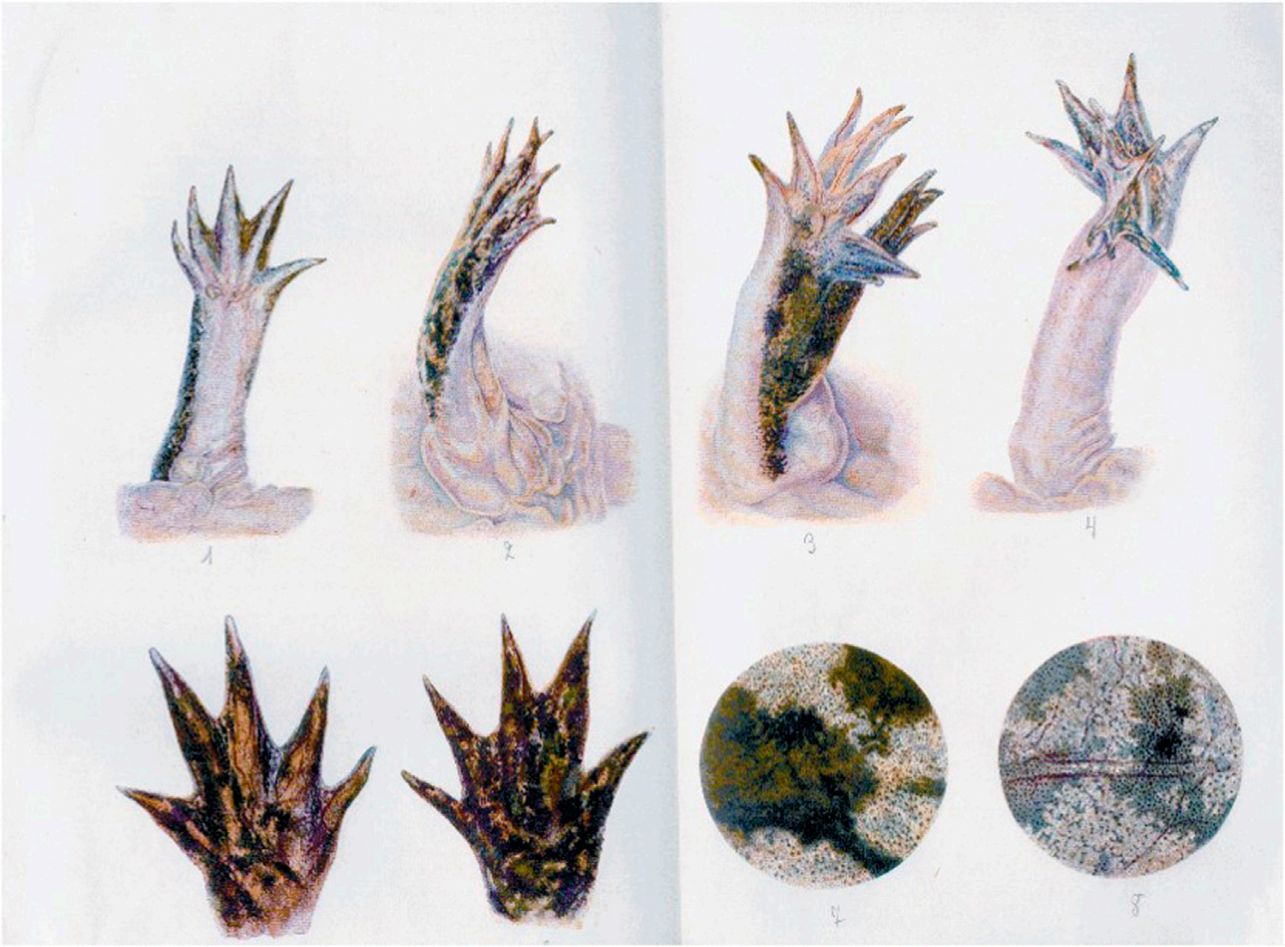
Illustration from Schaxel’s “Untersuchungen”. It shows the different ways in which he had used regeneration as a tool.

For his experiments, Schaxel would surgically remove organs and tissues (extirpation, amputation and excision), he would transplant them to different locations or he would keep them in artificial media as cultures (Schaxel 1921a: 17–18) (Figure 4). On a practical level, he was interested in the questions about the conditions for processes of regeneration after the loss of body parts and the causes that initiate, drive and end these processes (Schaxel 1921a: 16).

Schaxel published the first results of his research as a monograph and the first part of the “Untersuchungen zur Formbildung der Tiere”. Titled “Auffassungen und Erscheinungen der Regeneration”, this monograph was the first volume of the book series “Arbeiten auf dem Gebiet der experimentellen Biologie” edited by himself. Both the “Untersuchungen” and the “Arbeiten” did not see further publications. Schaxel would publish a number of related papers in various journals (Schaxel 1921b; 1922a; 1922b; 1922c; 1923)—including one on regeneration in insects (Schaxel and Adensamer 1923) until his political engagement caused a pause in his scientific research (Hopwood 1997; Reiß 2007). It was only in 1928, that he resumed to publish on his regeneration research, now together with a number of doctoral students (Schaxel 1928; Schaxel and Böhmel 1928; Schaxel and Haedeke 1928). He continued with his experiments after his escape from Germany to the Soviet Union (Schaxel 1934a, 1934b; Schaxel and Ivanova 1939; Schaxel 1940). He was one of the first individuals to be expelled from Jena university after the national socialist came to power in 1933 and left for a position at the Institute for Evolutionary Morphology and Palaeozoology at the Academy of Sciences of the Soviet Union. Here he would lead a laboratory for developmental mechanics (Reiß et al. 2008).

## Conclusion

Particularly in the axolotl, regeneration had an ambiguous status between question and tool, between epistemic thing and technical object. Researchers asked whether regeneration was the repetition of ontogenetic development or an entirely different process. Embryologists used it as a tool to understand regulation by repeatedly studying developmental events and processes on the macro level.

Together with the techniques of transplantation and extirpation—the practices of cut and paste—it became a powerful tool in the experimental systems of developmental biology. Regeneration research in the axolotl continued in the second half of the 20th century (Thornton 1968; Stocum 1995; Nye et al. 2003). Similar to many other fields in biology it was deeply transformed by the new methods of molecular biology (Geraudie and Ferretti 1998). It was especially with Elly Tanaka’s introduction of genetically modified axolotls in the 2000s (Sobkow et al. 2006) and the sequencing of the entire axolotl genome in 2018 (Nowoshilow et al. 2018) that the axolotl as a laboratory animal was put into an entirely different research context. The search for the molecular mechanisms of regeneration shifted attention from very basic questions of vertebrate development to the promises of regenerative medicine (McCusker and Gardiner 2011).

In many ways, regeneration research in the axolotl shows core aspects of the historical development of the life sciences and of the sciences in general—the advent of the experiment, the increase of publications and the European and then global circulation. Without a great discovery or a heated controversy—without the peaks Churchill warned about—the case of the axolotl helps to take a panoramatic view over time and space on the development of regeneration research. It highlights the availability of animals and infrastructure, the circulation of practices and the emergence of experimental systems with regeneration as epistemic thing and technical object in the wider experimental culture of experimental embryology. But the history of the axolotl is also special. Its long history in natural history and then as a living animal in zoos and laboratories sheds light on the long durée interconnectedness of research questions in particular animals. The fact that axolotls came to Europe in the context of imperial animal trade makes their transition into laboratory and experimental animals—their “generative entrenchment”—particularly visible.

## Footnotes

1 All translations are mine, except where indicated.

2 Constant Duméril published the work between 1834 and 1854 in nine volumes together with Gabriel Bibron (1805–1848). From volume seven onwards, Auguste Duméril is listed as the third co-author.

3 In contrast, Blumenbach is giving a physiological explanation based on his developmental thinking in his “Specimen physiologiae comparatae inter animantia calidi et frigidi sanguinis” (Blumenbach 1787) that Constant Duméril cites.

4 Even though there were already animals of the white color variety of the axolotl in Europe, it took until the beginning of the 20th century for them to become more widely available.

5 In contrast, the species' history in nature is not a success story. Today, only a few hundred individuals remain in its natural habitat in the canals of Xochimilco in Mexico City. The species is classified as critically endangered by the IUCN (Voss et al. 2015).
